# Effects of Different Processed Products of *Polygonum multiflorum* on the Liver

**DOI:** 10.1155/2020/5235271

**Published:** 2020-03-09

**Authors:** Ruo-Lan Li, Feng Gao, Shu-Ting Yan, Li Ou, Ming Li, Lin Chen, Pei-Feng Wei, Zong-Qiang Gao

**Affiliations:** ^1^College of Pharmacy, Shaanxi University of Chinese Medicine, Shiji Road, Qindu District, Xianyang, Shaanxi 712046, China; ^2^The third Department of bone, The Second Affiliated Hospital of Xi'an Jiaotong University, No. 157, Xiwu Road, Xincheng District, Xi'an City, Shaanxi 710004, China

## Abstract

**Objective:**

Based on in vitro and in vivo experimental studies, the changes of the main components of *Polygonum multiflorum* and different processed products and their effects on hepatotoxicity were investigated.

**Methods:**

The components of different processed products of *Polygonum multiflorum* were determined by HPLC. The effects of processed products of different processing time periods on HepG2 cells were detected by using cell count kit-8 and the apoptosis method; the effects of different processed products on the mouse liver were detected by reverse transcription polymerase chain reaction and immunohistochemistry.

**Results:**

With the extension of processing time, the contents of various chemical components in *Polygonum multiflorum* increased, while the content of stilbene glucoside decreased. The serum of *Polygonum multiflorum* group and different steaming time groups had obvious inhibitory effect on HepG2 cells. For normal mice, the toxicity of raw *Polygonum multiflorum* and processed products at different processing time periods had certain toxicity to liver and gradually decreased with the prolongation of processing time. For mice in the liver injury group, the therapeutic effect of raw *Polygonum multiflorum* and processed products at different processing time periods was not obvious, but there is a trend of treatment.

**Conclusion:**

The content of the main components in Radix *Polygonum multiflorum* can be affected by processing time; stilbene glycoside may be the main component leading to liver injury. The degree of liver injury caused by Radix *Polygonum multiflorum* is negatively correlated with processing time.

## 1. Introduction

*Polygonum multiflorum* Thunb. is the root of *Polygonum multiflorum*. It has the effect of moistening bowel, relieving stool, detoxification, cutting malaria, hair of hair, nourishing blood, and so on [1]. It is a good product to prolong life and improve blood circulation. Combined with a variety of tonic drugs, it can give full play to the efficacy of drugs. Modern studies have shown that *Polygonum multiflorum* Thunb. has the functions of delaying senility, regulating blood lipids, and antiatherosclerosis. It has been widely used in Europe and the United States as herbals and dietary supplements [2]. The chemical constituents of *Polygonum multiflorum* mainly include stilbene glycosides, terpenoids, and phospholipids. It was speculated that the main toxicants might be stilbenes [3] or anthraquinones [4], which are two major constituents of *Polygonum multiflorum*.

Nevertheless, series of reports of adverse hepatotoxic effects induced by *Polygonum multiflorum* [5] have been occasionally reported in recent years. The US National Library of Medicine (USNLoM) has independently included the drug-induced liver damage of *Polygonum multiflorum* as a special topic in its database. Relevant domestic research results show that the incidents of liver injury caused by *Polygonum multiflorum* rank first in Chinese medicinal materials, which has aroused close attention of scholars at home and abroad to the safety of *Polygonum multiflorum* [6]. In recent years, there have been more and more reports on liver injury caused by *Polygonum multiflorum* Thunb. and more experimental studies have been carried out. At present, a large number of scholars have carried out research on the processing technology, chemical composition, pharmacological effect, and so on in Pharmacopoeia [7]. The processing materials of *Polygonum multiflorum* prepared in 2015 edition of Chinese Pharmacopoeia are recorded: every 100 kg of *Polygonum multiflorum* tablets, 10 kg of black beans are used, and the therapeutic effect of *Polygonum multiflorum* can be enhanced and changed after being prepared with black bean juice [8]. Modern research shows that the toxicity of *Polygonum multiflorum* Thunb. can be significantly reduced after processing. The inconsistent processing parameters directly lead to the change of the composition and content of the components in *Polygonum multiflorum* Thunb., thus producing different degrees of toxic and side effects [3]. In order to alleviate and avoid the hepatic injury caused by *Polygonum multiflorum* Thunb., scholars proposed to reduce the toxicity of *Polygonum multiflorum* Thunb. from the perspective of processing. Thus, it can be seen the processing of Chinese herbal medicines is important because it has been shown to decrease toxicity and alter the therapeutic efficacy of the extracts [3]. Based on the theory of “time-effect-toxicity,” this experiment will continue to study the degree of liver damage caused by different processed products of Radix *Polygonum multiflorum* from the perspective of processing time.

## 2. Materials and Methods

### 2.1. Materials, Chemicals, and Animals

The raw roots of *Polygonum multiflorum* Thunb. (NT. 20170628) were purchased from Xingshengde Pharmaceutical Co., Ltd. (Shaanxi, China), identified by Professor Benxiang Hu (Shaanxi University of Chinese Medicine) and verified to have met the standards specified by Chinese Pharmacopoeia. HPLC-grade ethyl acetate, N-butanol, formic acid, acetonitrile, and other reagents used in this study were purchased from Chromatography Science Instrument Co., Ltd. (Shenyang, China). Ultrapure distilled water was prepared from a Milli-Q purification system in the lab. Annexin V-FITC/PI was purchased from Nanjing Jiancheng Institute of Bioengineering Institute (Nanjing, China). Cysteine aspartic acid specific protease-3 (caspase-3), B cell lymphoma-2 (Bcl-2), and nuclear factor kappa-B (NF-*κ*B) antibody were purchased from Wan Lei Biotechnology Co., Ltd. (Shenyang, China). HRP-conjugated Goat Anti-Rabbit IgG antibody was purchased from Thermo Fisher Scientific. Alanine aminotransferase (ALT), aspartate aminotransferase (AST), and alkaline phosphatase (ALP) were purchased from Wan Lei Biotechnology Co., Ltd. (Shenyang, China).

Six-week-old male Sprague-Dawley (SD) rats with an average body weight of 200–220 g and four-week-old male specific-pathogen-free mice with an average body weight of 20–22 g were purchased from Sichuan experimental animal quality testing center (Sichuan, China). All animals were acclimatized in an air-conditioned room at 25 ± 1°C, a relative humidity of 50 to 60%, a 12/12 h light/dark cycle, free to standard laboratory feed, and tap water for 7 days before experiments. All animal experiments were conducted in accordance with the British Animal (scientific procedure) Act, 1986, and related guidelines, Council Directive of the European Communities of 24 November 1986 (86/609/EEC), and the National Institutes of Health (NIH publication 8023, revision 1978).

### 2.2. HPLC Analysis of Raw and Processed *Polygonum multiflorum* Thunb

The standard substances of emodin, emodin methyl ether, rhein, gallic acid, and stilbene glycoside were precisely weighed and dissolved in methanol to prepare standard solutions with different concentration gradients. The raw and the processed products of steamed 8 h, 16 h, 32 h, 48 h, and 72 h *Polygonum multiflorum* Thunb. powder were extracted with 80% EtOH under reflux for 1 h and filtered. The filtrate was evaporated and dissolved in 80% methanol to 10 ml. The test solutions were successfully obtained. Standard stock solutions and test solutions were HPLC; analyses were performed on UltiMate 3000 HPLC [9] (Thermo Fiser Scientific, Shanghai, China); the separation was achieved using a Hypersil GOLD aQ C_18_ column (5 *μ*m, 250 mm × 4.6 mm). The gradient mobile phase consists of methanol (A) and water containing 0.1% formic acid (B) delivered at a flow rate of 1.0 mL·min^−1^. The gradient was programmed as follows: 0–19 min, 10%–36% A, 90%–63% B; 19–30 min, 36%–71% A, 63%–29% B; 30–50 min, 71%–100% A, 29%–0% B; 50–60 min, 100% A. The column temperature was maintained at 35°C, and detection was carried out at 254 nm. The injection volume was 10 *μ*L.

### 2.3. Cell Survival Rate and Apoptotic Rate

The Radix *Polygonum multiflorum* was processed according to the method of the Pharmacopoeia 2015 (steaming method). The Radix *Polygonum multiflorum* was divided into different sizes. Mixed with clear water, it was moistened thoroughly and placed in a suitable steaming container. Steam was heated for 8 h, 16 h, 32 h, 48 h, and 72 h, then taken out, and cooled slightly. The steamed liquid was mixed with medicinal materials and then dried to 60%. 100 kg *Polygonum multiflorum* Thunb. was treated with 20–30 kg clear water.

The processed products of Radix *Polygonum multiflorum* with different processing time were soaked in 5 times of cold water for 1 hour, then boiled and cooked for 30 minutes, and then filtered. Three times of cold water was added to the filter residue, decocted for 20 minutes, filtered, and combined with two filters to concentrate and reserve. Male mice were random divided into seven groups (6 animals/group). The mice of the first group (control group) were fed with water only. The mice of Groups 2 to 7 were treated with the raw and the steamed 8 h, 16 h, 32 h, 4 8h, and 72 h *Polygonum multiflorum* powder as described above at the doses of 12 g/kg of the body weight per day od (counted on the quantity of crude material). The water and extracts were given intragastrically. The dosage was given twice a day for 7 consecutive days. The blood was collected by negative pressure tube of abdominal aorta after the last 2 hours of gastric perfusion. The blood was placed for 30 minutes and centrifuged for 10 minutes at 3000 rpm. The serum was inactivated at 37°C for 30 minutes, filtered, and stored at−20°C for reserve.

Human HepG2 hepatoma cell was cultured in Dulbecco's modified Eagle's medium (DMEM) containing 10% fetal bovine serum (FBS) supplemented with penicillin (100 U/mL) and streptomycin (100 *μ*g/mL). The cells were maintained at 37°C in a humidified atmosphere of 5% CO_2_ and 95% air.

Cell inhibitory assays were performed using a Cell Counting Kit-8 (CCK-8; BOSTER, Wuhan, China) according to the manufacturer's protocol. When the adherent cells in culture flask grew to logarithmic phase, they were cultured in a 96-well plate at a concentration of 1 × 10^5^ cells/mL for 24 h. Then, medium containing 15% rat serum containing different processed products of *Polygonum multiflorum* and blank rat serum was added to well; each group had eight wells. 24 hours later, the CCK-8 was added to each well, and the plates were incubated for 2 h at 37°C in the condition of avoiding light. Cell inhibition was determined by measuring the absorbance at 450 nm by using an ELX808-IU microplate reader (Biotek Instruments, USA).

The apoptotic rate was detected by flow cytometry using the Annexin V-FITC/propidium iodide (PI) method. HepG2 cells (2.5 × 10^5^ cells/mL) were seeded in a 6-well plate for 24 h. Next, the original medium was abandoned and the medium containing 15% rat serum containing different processed products of *Polygonum multiflorum* and blank rat serum for 24 h was added. The cells were then treated with trypsin without EDTA and collected by centrifugation at 2000 rpm for 5 min. After washing with PBS, cells were then double-stained using an Annexin V-FITC apoptosis detection kit [10]. According to the manufacturer's protocol, cells resuspended in Annexin V-FITC binding buffer were incubated with Annexin V-FITC for 15 min at room temperature in the dark and were then incubated with PI. Samples were analyzed with a flow cytometer.

### 2.4. Animal Experiments

Male SPF mice were divided into seven groups: control group (A, 10 mice), unprocessed Radix *Polygonum multiflorum* (B, 10 mice), and five groups of Radix *Polygonum multiflorum* with different processing time groups (C-G). Treatment was administered by oral gavage once a day for 28 consecutive days. The mice of control group were given water while the mice of other groups fed with different processed products of *Polygonum multiflorum* to 12 g/kg of raw materials, respectively.

Male SPF mice were divided into nine groups: control group (A, 10 mice), liver injury model group (B, 10 mice), positive control group (C, 10 mice), unprocessed Radix *Polygonum multiflorum* (D, 10 mice), and five groups of raw Radix *Polygonum multiflorum* with different processing time groups (E-I). In addition to the blank group, mice in each group were subcutaneously injected with 10% CCl_4_ diluted with peanut oil, and the models were made every two days. After the first establishment of the model, the rats were given intragastric administration at a dose of 6 g/kg. The blank group and the model group were given the same amount of normal saline, and the positive group was given bifendate dropping pills (200 mg/kg).

Ten hours later, blood samples were collected into tubes and then centrifuged for ten minutes at 3000 rpm to afford serums. The serums were stored at −20°C for further analysis. ALT, AST, and ALP were measured in the serums after one freeze-thaw cycle by using the enzyme-labeled instrument assay. The liver tissues were flushed with normal saline to remove residual blood and immersed in 4% neutral-buffered formaldehyde (paraformaldehyde solution) immediately before being stored at −80°C. Part of the liver tissue was embedded in paraffin, sectioned (thickness of 4 *μ*m), and stained with H&E.

### 2.5. Quantitative Real-Time Polymerase Chain Reaction (RT-qPCR) Analysis

CYP3A, CYP1A2, and CYP2E1 in the liver were analyzed using RT-qPCR. PCR was performed using 100 ng of cDNA. The PCR conditions consisted of AmpliTaq Gold Enzyme activation for 10 min at 95°C, followed by 40 cycles of heating to 95°C for 15°s and cooling to 60°C for 1 min. The mRNA levels were normalized to those of *ß*-actin. The following primers were used for PCR: *ß*-actin: sense, 5′-GGAGATTACTGCCCTGGCTCCTAGC-3′; antisense, 5′-GGC-CGGACTCATCGTACTCCTGCTT-3′. CYP3A: sense, 5′-GGCAAGCCTGTTACTA-T-GAAAG-3′; antisense, 5′-ACTGAGAAGAGCAAAGGATCAA-3′. CYP1A2: sense, 5′-ATCCTGGAGATCTACCGATACA-3′; antisense, 5′-TATGTAGATACAGC-GCTCCTTG-3′. CYP2E1: sense, 5′-CCAACTCTGGACTCCCTTTTAT-3′; antisense, 5′-ACGCCTTGAAATAGTCACTGTA-3′.

### 2.6. Immunohistochemistry

For immunohistochemistry, liver tissue sections were prepared for blocking and incubating with antibody of caspase-3, Bcl-2, and NF-*κ*B. In chromogenic experiments, endogenous peroxidase was blocked using 3% hydrogen peroxidase for 10 minutes and protein blocking was performed using TBS-Tween 1% BSA for 20 minutes. The first antibody was incubated overnight at 4°C, then Goat Anti-Rabbit (IgG) was incubated at 37°C for 30 min, the second antibody was added dropwise the next day, DAB was stained for 5 min, and hematoxylin was redyed for 2 min and then analyzed under a phase-contrast microscope.

### 2.7. Statistical Analysis

Data analysis was performed with SPSS (SPSS version 14.0) software. In addition, all statistical column charts were drawn with Origin 8.0. The quantitative data were presented as mean ± standard deviation (±SD). The variance of more than two sets of data was determined by one-way analysis of variance (one-way ANOVA), and multiple comparisons were performed by Duncan's multiple-range comparisons. Two-tailed Student's *t*-test was used to perform the statistical analysis of two sets of data.

**Figure 1:**
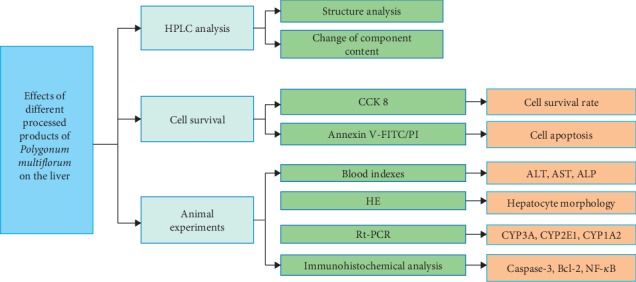
Experimental research process.

We summarized the experimental process performed in this article, as shown in Figure 1.

## 3. Results

### 3.1. Study on the Content of Different Processed Products of *Polygonum multiflorum*

**Figure 2:**
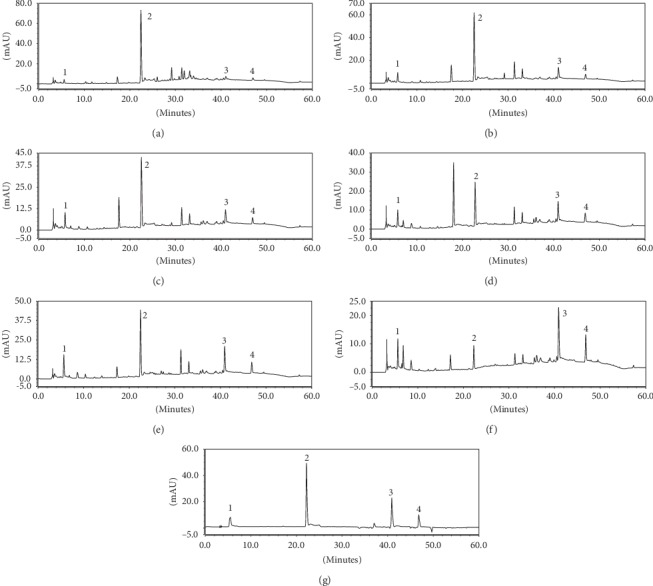
Chromatogram of four components of several processed products of *Polygonum multiflorum.* (a) Raw Radix *Polygonum multiflorum* (b) steamed for 8 h, (c) steamed for 16 h, (d) steamed for 32 h, (e) steamed for 48 h, (f) steamed for 72 h, and (g) mixed standard).

The content of 3,4,5-trihydroxybenzoic acid (1), emodin (3), and 1,8-dihydroxy-3-methoxy-6-methylanthraquinone emodin 3-methyl ether (4) in processed *Polygonum multiflorum* was lower than that in raw *Polygonum multiflorum* by HPLC. The content of various components in different processing time groups increased with the increase of processing time, but the content of 2,3,5,4′-tetrahydroxystilbene-2-o-*β*-D-glucoside (2) decreased with the increase of processing time, as shown in Figure 2.

### 3.2. Cell Survival Rate and Apoptotic Rate

**Figure 3:**
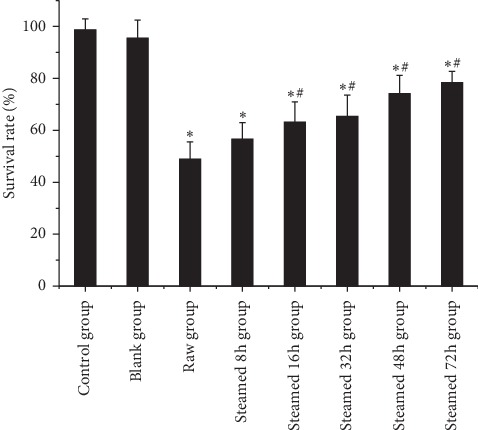
Cell survival rate of different processing time groups of *Polygonum multiflorum.* Results were presented as mean ± SD. *∗P* < 0.05 versus blank group. #*P* < 0.05 versus raw group.

As shown in Figure 3, fetal bovine serum as control group, the drug containing serum of the raw Radix *Polygonum multiflorum* group, and different processing time groups of *Polygonum multiflorum* obviously inhibited HepG2 cells. The cell survival rate increased with the increase of processing time and the survival rate of cells was the lowest in the steamed 8 h group and the highest in the 72 h group. Compared with the blank group, the cell survival rate of the treated group was statistically significant.

**Figure 4:**
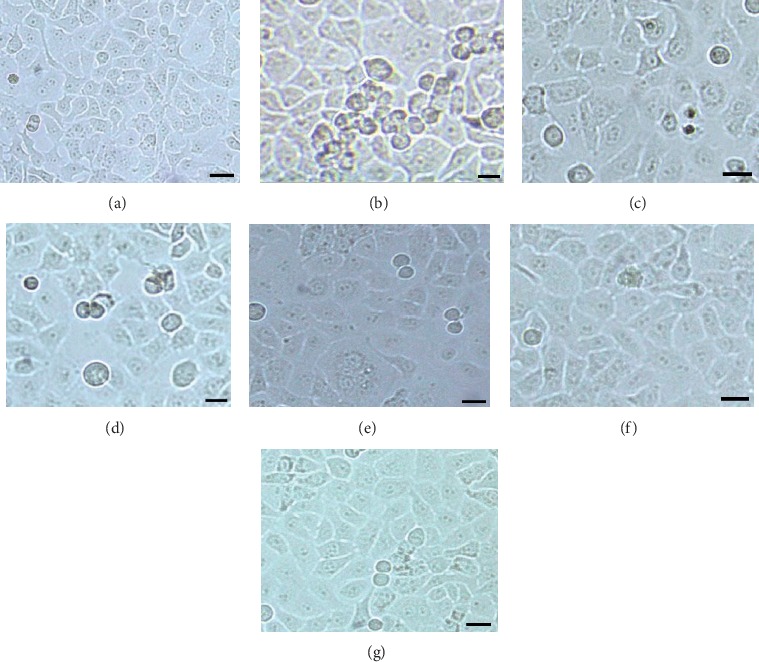
Effect of serum containing medicine on morphology of HepG2 cells. (a) blank group; (b) raw Radix *Polygonum multiflorum* group; (c)–(g) 8 h, 16 h, 32 h, 48 h, and 72 h steaming group.

As shown in Figure 4, the HepG2 cells cultured for 24 hours after administration were observed under 20 times microscope. The cells in the blank group grew fusiform and adhered to the wall without aging or floating dead cells. Compared with the blank group, most of the cells in the raw Radix *Polygonum multiflorum* group and the steamed 8h group broke and necrotized after 24 hours; the cell morphology changed. HepG2 cells in the 16 h, 32 h, 48 h, and 72 h groups were necrotic and ruptured in varying degrees, but the degree of injury showed a decreasing trend, indicating that the steamed products of *Polygonum multiflorum* would reduce the damage to cells with the prolongation of processing time.

**Figure 5:**
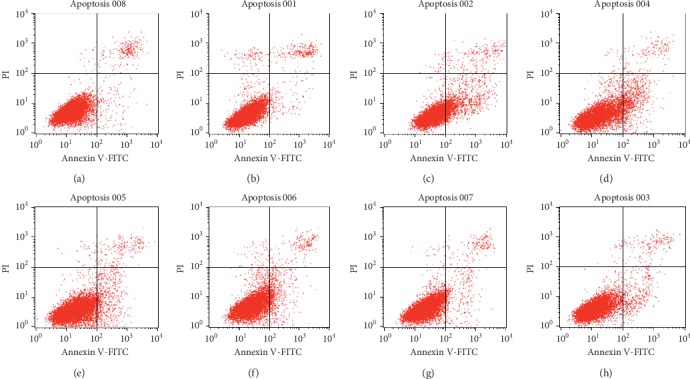
Effect of serum containing medicine on HepG2 cell apoptosis. (a) Control group; (b) blank group; (c) Radix *Polygonum multiflorum* group; (d)–(h) 8 h, 16 h, 32 h, 48 h, and 72 h steaming group.

As shown in Figure 5, fetal bovine serum as control group, after 24 hours of drug administration and culture, the cells in each group were detected by annexin v-pi kit. The results showed that the cells in each group had apoptosis and necrosis in different degrees in the early and late stages, and the apoptosis rate decreased gradually with the increase of processing time; the cells in the raw Radix *Polygonum multiflorum* group and steamed groups mainly appeared in AV+ and PI− areas, indicating that the cells in those groups containing the drug serum can induce HepG2 cell apoptosis. Compared with the blank group, the apoptosis rate was statistically different, and the steamed group was also statistically different from the raw Radix *Polygonum multiflorum* group.

### 3.3. Blood Biochemical Indexes Analysis

**Figure 6:**
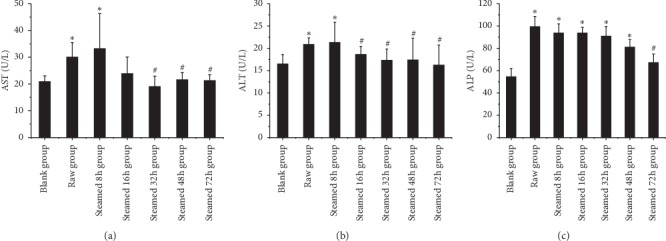
The effect of liver injury on blood biochemical indexes. Results were presented as mean ± SD. *∗P* < 0.05 versus blank group. #*P* < 0.05 versus raw group.

**Figure 7:**
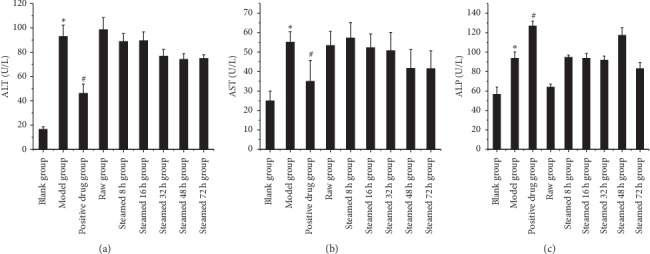
The effect of liver protection on blood biochemical indexes. Results were presented as mean ± SD. *∗P* < 0.05 versus blank group. #*P* < 0.05 versus model group.

**Figure 8:**
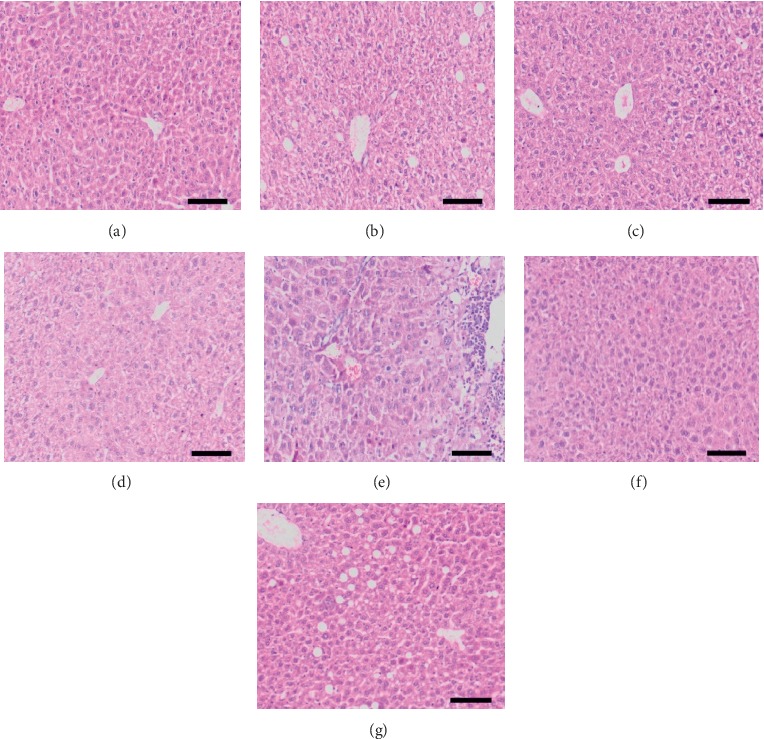
Pathological sections of liver tissue of mice in liver injury group. (a) Blank group; (b) Radix *Polygonum multiflorum* group; (c)–(g) 8 h, 16 h, 32 h, 48 h, and 72 h steaming group.

As shown in Figure 6, blood biochemical indexes of liver injury group showed that ALT and AST of the raw Radix *Polygonum multiflorum* group and steamed 8 h group were statistically significant compared with blank group. The steamed 16 h, 32 h, 48 h, and 72 h groups were statistically different from the raw Radix *Polygonum multiflorum.* ALP of the raw Radix *Polygonum multiflorum* group and steamed 8 h, 16 h, 32 h, and 48 h groups was statistically significant compared with blank group. The steamed 72 h group was statistically significant compared with the raw Radix *Polygonum multiflorum.* As shown in Figure 7, the blood biochemical indexes of liver protection group showed that there were significant differences in ALT, AST, and ALP between model group and blank group, and there were no significant differences in ALT, AST, and ALP between different processing time groups and model group. As shown in Figure 8, the HE staining pathological section of liver in the liver injury group showed that the hepatocytes in the blank group were normal and the liver tissue structure was clear and complete. The hepatocytes in the raw Radix *Polygonum multiflorum* group and the steamed 8 h group showed that large area swelling, a small amount of necrosis, a small amount of cytoplasmic reduction or loss, inflammatory cell infiltration, and a small amount of blood cells in the stroma were leached and red stained. The hepatocytes in the groups of steamed 16 h, 32 h, 48 h, and 72 h after steaming were slightly swollen and necrotic. It can be seen that the inflammation and necrotic symptoms were relieved as the processing time increased.

**Figure 9:**
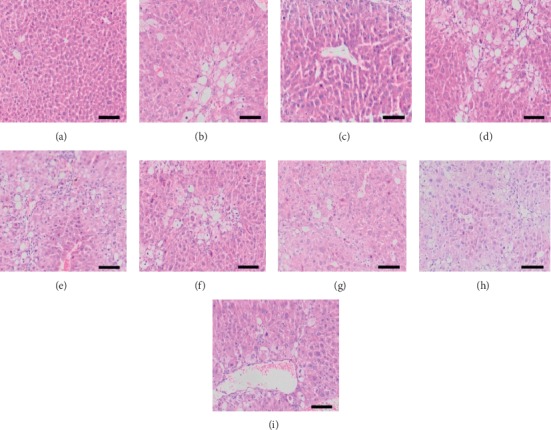
Pathological section of liver tissue of mice in liver protection group. (a) Blank group; (b) model group; (c) positive drug group (d) Radix *Polygonum multiflorum* group; (e)–(i) 8 h, 16 h, 32 h, 48 h, and 72 h steaming group.

The pathological section of liver in the liver protection group showed that the liver in the blank group was normal, without swelling or necrosis. In the model group, the liver structure was damaged, the nucleus became larger, and the structure of liver lobules and the arrangement of liver cords were disordered. Compared with the model group, the swelling and necrosis of hepatocytes in the positive drug group were significantly improved, while those in the raw Radix *Polygonum multiflorum* group, the steamed group for 8 h, 16 h, 23 h, 48 h, and 72 h, were not significantly improved, as shown in Figure 9.

### 3.4. Analysis of the Expression of CYP3A, CYP1A2, and CYP2E1

**Figure 10:**
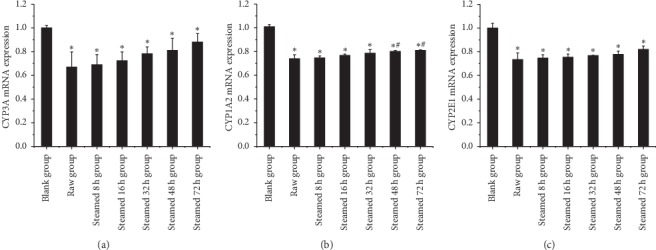
Effect of liver injury group on three CYP subtype enzyme indexes. Results were presented as mean ± SD. *∗P* < 0.05 versus blank group. #*P* < 0.05 versus raw group.

**Figure 11:**
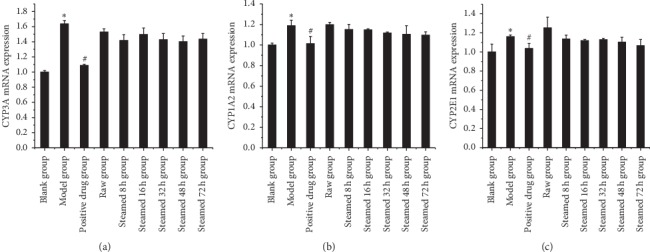
Effect of liver protection group on three CYP subtype enzyme indexes. Results were presented as mean ± SD. *∗P* < 0.05 versus blank group. #*P* < 0.05 versus model group.

As shown in Figure 10, RT-PCR results of liver injury group showed that the expression of CYP3A, CYP1A2, and CYP2E1 in the raw Radix *Polygonum multiflorum* group and steaming group for 8 h, 16 h, 32 h, 48 h, and 72 h was significantly different from that in blank group; with the increase of processing time, the expression of three CYP enzymes in different processing time groups was gradually increasing. The results of liver protection group showed that there was significant difference in the expression of three CYP subtypes between the model group and the blank group. The relative expression of three subtypes in different processing time groups showed a downward trend, but there was no statistical difference between the model group and the model group, indicating that the drug had no significant protective effect on the liver, as shown in Figure 11.

### 3.5. Immunohistochemical Analysis of Caspase-3, Bcl-2, and NF-*κ*B

**Figure 12:**
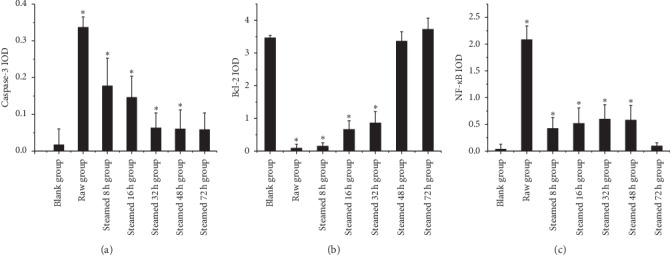
Expression of three indexes of immunohistochemistry in liver injury group. Results were presented as mean ± SD. *∗P* < 0.05 versus blank group.

As shown in Figure 12, in the liver injury group, the results of immunohistochemistry showed that the expression of Caspase-3 and NF-*κ*B protein in the steamed 8 h, 16 h, 32 h, and 48 h groups were statistically significant compared with the blank group. The expression of Bcl-2 protein in the steamed 8 h, 16 h, and 32 h was statistically significant compared with the blank group. The expression of caspase-3 and NF-*κ*B decreased and Bcl-2 increased in different processing time groups.

**Figure 13:**
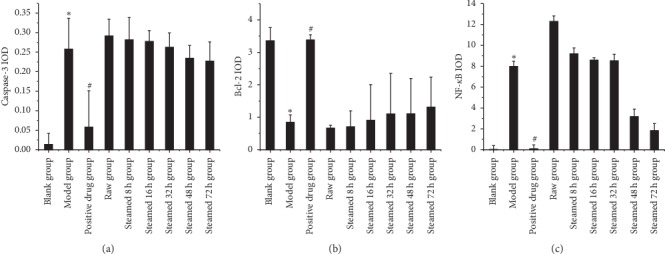
Expression of three indexes of immunohistochemistry in liver protection group. Results were presented as mean ± SD. *∗P* < 0.05 versus blank group. #*P* < 0.05 versus model group.

The results of liver protection group showed that the expression of caspase-3, Bcl-2, and NF-*κ*B in the model group was significantly different from that in the blank group, and the positive drug group was also statistically significant compared with the model group. However, there was no significant difference in the expression of various indexes between different processing time groups and raw *Polygonum multiflorum* group compared with the model group, as shown in Figure 13.

## 4. Discussion

The frequency of application of *Polygonum multiflorum* in clinical medicine is relatively high, and there are many reports about its liver injury [11]. In recent years, most of the reports about liver injury at home and abroad are about acute liver cell injury [12]. Drug-induced liver injury may lead to hepatocyte apoptosis and necrosis (71.9%), massive inflammatory cell infiltration (93.8%), punctate necrosis (96.9%), and other pathological manifestations [13]. At present, most of the researches on liver injury are based on the detection of pathological tissue, biochemical indexes, and the expression of related protein factors in animal model and in vitro cell model. HepG2, a human hepatoma cell line, has many characteristics and functions of human normal hepatocytes. It retains a relatively complete bio transferase system, has high sensitivity, and is more stable and mature than primary hepatocytes. It is considered to be one of the ideal models for screening drug hepatotoxicity in vitro [14]. The liver is the main organ of metabolism and one of the target organs of drug toxicity. When drugs enter the body, the metabolism of endogenous substances such as drug metabolizing enzymes has a certain impact on drug metabolism [15]. Toxic components or substances cause abnormal liver function and corresponding protein molecules and drug metabolizing enzyme mRNA expression change [16]. After liver cells are damaged, AST and ALT will reduce the enzyme activity of patients. For patients with severe liver injury, AST and ALT will directly penetrate into the blood, so as to improve the enzyme content in the blood [17]. We regard the elevation of serum AST and ALT as a classical index of clinical hepatocyte injury.

Clarifying the metabolic pathway of drugs in vivo is an important way to study drug liver injury [18]. Apoptosis is mainly regulated by mitochondrial pathway and membrane receptor pathway. Mitochondrial pathway is also known as internal regulatory pathway, which is mainly regulated by Bcl-2 family [19]. NF-*κ*B is an important nuclear transcription factor, which plays an important role in the process of apoptosis. NF-*κ*B can mediate multiple signal transduction pathways related to cell differentiation and immune response. NF-*κ*B activation can regulate the transcription of Bcl-2 gene and upregulate the expression of Bcl-2 protein after entering the nucleus [20] and then inhibition of cell apoptosis. When the expression of Bcl-2 protein in mitochondria decreased, the permeability of outer membrane increased, the cell membrane perforated, and the cytochrome C in mitochondria was released, which activated the downstream caspase protein family, thus promoting the occurrence of apoptosis [21]. It has been reported that Bcl-2 can act as a direct substrate of caspase-3, and the N-terminal variable loop of Bcl-2 protein can be cut by caspase-3 at asp34 and split into Bax-like apoptotic fragments, which can accelerate the process of apoptosis [22]. This step has been proved to be one of the most critical speed limiting steps in apoptosis signal transduction [23]. In addition, Bcl-2 can directly bind with apoptotic protein active factor-1 (Apaf-1), forming a complex to block the initiation of caspase activation [24]. Caspases protein is the main promoter of apoptosis, and all the signal pathways of apoptosis are ultimately completed by cascade reaction of caspases family protease [25]. Caspase-3 is one of the key points. It is activated in the endogenous pathway and leads to the cleavage of poly ADP ribose polymerase (PARP) and apoptosis. Therefore, caspase-3 plays a key role in the initiation and execution of apoptosis [26].

Cytochrome P450 enzyme (CYP450) is an important phase I drug metabolism enzyme system in human body, which mainly exists in liver microparticles and widely participates in the metabolism of endogenous and exogenous compounds [27]. The interaction between drugs is mainly inhibited and induced by the activity of metabolic enzymes [26, 28]. CYP3A is one of the most important subtypes of CYP450 enzymes, accounting for 25% of the total CYP450 enzymes in adult liver [29]. It is the isoenzyme with the highest content and the most metabolic substrate in CYP450 enzyme system, which is related to the interaction of various drugs [28]. CYP1A2 accounts for 13% of P450 in human liver, and most chemicals and poisons are metabolized by CYP1A2 [30]. Although CYP2E1 accounts for 7% of CYP450 in liver, it is the metabolic enzyme of many small organic compounds and drugs [31].

The RT-PCR results in this article (Figures 10 and 11) show that different processed products of *Polygonum multiflorum* can reduce the expression of CYP1A2, CYP3A, and CYP2E1 in normal rat liver but have no obvious effect on the injured rat liver. Similarly, the results of immunohistochemistry (Figures 12 and 13) show that different processed products of *Polygonum multiflorum* can increase the expression of caspase-3 and NF-*κ*B in normal rat liver and reduce the expression of Bcl-2. There was no effect on the injured rat liver. In conclusion, the toxicity of *Polygonum multiflorum* is weakened after processing, and there is a negative correlation between processing time and toxicity; that is, the longer the processing time is, the less the toxicity of *Polygonum multiflorum* is. The toxic mechanism of *Polygonum multiflorum* on liver is different from that of CCl4, and its protective effect on liver is relatively less obvious. In this paper, the correlation of “time-effect-poison” of raw *Polygonum multiflorum* and processed products at different processing times was studied in depth from the aspects of liver pathology, cell apoptosis, and drug metabolism enzymes. In the follow-up study of liver injury caused by *Polygonum multiflorum*, we should further explore the specific pathway and related target genes of its role, clarify the specific mechanism of liver injury caused by *Polygonum multiflorum*, provide theoretical support for clinical correct drug use, reduce safety risks, and make contributions to human health.

## Data Availability

The data used to support the findings of this study are included within the article.

## References

[1] Lin L., Ni B., Lin H. (2015). Traditional usages, botany, phytochemistry, pharmacology and toxicology of *Polygonum multiflorum* Thunb: a review. *Journal of Ethnopharmacology*.

[2] Li C., Niu M., Bai Z. (2017). Screening for main components associated with the idiosyncratic hepatotoxicity of a tonic herb, *Polygonum multiflorum*. *Frontiers of Medicine*.

[3] Wu X., Chen X., Huang Q., Fang D., Li G., Zhang G. (2012). Toxicity of raw and processed roots of *Polygonum multiflorum*. *Fitoterapia*.

[4] Park G. J.-H., Mann S. P., Ngu M. C. (2001). Acute hepatitis induced by Shou-Wu-Pian, a herbal product derived from *Polygonum multiflorum*. *Journal of Gastroenterology and Hepatology*.

[5] Lei X., Chen J., Ren J. (2015). Liver damage associated with *Polygonum multiflorum* Thunb: a systematic review of case reports and case series. *Evidence-Based Complementary and Alternative Medicine*.

[6] Ng K. Y., Cheng C. L., Xu H. X. (2009). Safety issues of Chinese medicine:a review of intoxication cases in Hong Kong. *Chinese Herbal Medicines*.

[7] Lv G., Lou Z., Chen S. (2011). Pharmacokinetics and tissue distribution of 2,3,5,4′-tetrahydroxystilbene-2-O-*β*-d-glucoside from traditional Chinese medicine *Polygonum multiflorum* following oral administration to rats. *Journal of Ethnopharmacology*.

[8] Sun Y. N., Cui L., Li W. (2013). Promotion effect of constituents from the root of *Polygonum multiflorum* on hair growth. *Bioorganic & Medicinal Chemistry Letters*.

[9] Huang Z., Huang X., Liu H. Rapid determination of ferulic acid in three kinds of Chinese herbs by direct analysis in real‐time mass spectrometry. *Separation Science Plus*.

[10] Yan H., Xue G., Mei Q. (2009). Repression of the miR-17-92 cluster by p53 has an important function in hypoxia-induced apoptosis. *The EMBO Journal*.

[11] Jung K. A., Min H. J., Yoo S. S. (2011). Drug-induced liver injury: twenty five cases of acute hepatitis following ingestion of *Polygonum multiflorum* Thunb. *Gut and Liver*.

[12] Dong H., Slain D., Cheng J., Ma W., Liang W. (2014). Eighteen cases of liver injury following ingestion of *Polygonum multiflorum*. *Complementary Therapies in Medicine*.

[13] Grattagliano I., Bonfrate L., Diogo C. V., Wang H. H., Wang D. Q., Portincasa P. (2009). Biochemical mechanisms in drug-induced liver injury: certainties and doubts. *World Journal of Gastroenterology*.

[14] Yano A., Oda S., Fukami T., Nakajima M., Yokoi T. (2014). Development of a cell-based assay system considering drug metabolism and immune- and inflammatory-related factors for the risk assessment of drug-induced liver injury. *Toxicology Letters*.

[15] Dallmann A., Ince I., Coboeken K., Eissing T. (2018). A physiologically based pharmacokinetic model for pregnant women to predict the pharmacokinetics of drugs metabolized via several enzymatic pathways. *Clinical Pharmacokinetics*.

[16] Ota T., Kamada Y., Hayashida M. (2015). Combination analysis in genetic polymorphisms of drug-metabolizing enzymes CYP1A2, CYP2C9, CYP2C19, CYP2D6 and CYP3A5 in the Japanese population. *International Journal of Medical Sciences*.

[17] Cho J.-H., Oh D.-S., Hong S.-H. (2017). A nationwide study of the incidence rate of herb-induced liver injury in Korea. *Archives of Toxicology*.

[18] Takakusa H., Masumoto H., Mitsuru A., Okazaki O., Sudo K. (2008). Markers of electrophilic stress caused by chemically reactive metabolites in human hepatocytes. *Drug Metabolism and Disposition*.

[19] Pu X., Storr S. J., Zhang Y. (2017). Caspase-3 and caspase-8 expression in breast cancer: caspase-3 is associated with survival. *Apoptosis*.

[20] Neuzil J., Wang X.-F., Dong L.-F. (2006). Molecular mechanism of “mitocan”-induced apoptosis in cancer cells epitomizes the multiple roles of reactive oxygen species and bcl-2 family proteins. *FEBS Letters*.

[21] Kirsch D. G., Doseff A., Chau B. N. (1999). Caspase-3-dependent cleavage of bcl-2 promotes release of cytochrome c. *Journal of Biological Chemistry*.

[22] Zhang J., Xia Y., Xiaomind Z. (2016). Propofol suppressed hypoxia/reoxygenation-induced apoptosis in HBVSMC by regulation of the expression of bcl-2, Bax, Caspase3, Kir6.1, and p-JNK. *Oxidative Medicine and Cellular Longevity*.

[23] Thornberry N. A. (1998). Caspases: enemies within. *Science*.

[24] Zhang K., Ge Z., Fu L. (2018). Qilin pills alleviate oligoasthenospermia by inhibiting Bax-caspase-9 apoptosis pathway in the testes of model rats. *Oncotarget*.

[25] Julien O., Wells J. A. (2017). Caspases and their substrates. *Cell Death and Differentiation*.

[26] Behrendorff J. B. Y. H., Huang W., Gillam E. M. J. (2015). Directed evolution of cytochrome P450 enzymes for biocatalysis: exploiting the catalytic versatility of enzymes with relaxed substrate specificity. *Biochemical Journal*.

[27] Kato M., Chiba K., Ito T., Koue T., Sugiyama Y. (2010). Prediction of interindividual variability in pharmacokinetics for CYP3A4 substrates in humans. *Drug Metabolism and Pharmacokinetics*.

[28] Tostmann A., Boeree M. J., Aarnoutse R. E. (2008). Antituberculosis drug-induced hepatotoxicity: concise up-to-date review. *Journal of Gastroenterology*.

[29] Ding Y., Zhang T., Tao J.-s., Zhang L.-y., Shi J.-r., Ji G. (2013). Potential hepatotoxicity of geniposide, the major iridoid glycoside in dried ripe fruits of *Gardenia jasminoides* (Zhi-zi). *Natural Product Research*.

[30] Ryu S.-D., Chung W.-G. (2003). Induction of the procarcinogen-activating CYP1A2 by a herbal dietary supplement in rats and humans. *Food and Chemical Toxicology*.

[31] Schwab M. (2013). Cytochrome P450 enzymes in drug metabolism: regulation of gene expression, enzyme activities, and impact of genetic variation. *Pharmacology & Therapeutics*.

